# Lipid levels and low back pain risk: A two-sample mendelian randomization study

**DOI:** 10.1371/journal.pone.0304280

**Published:** 2024-07-11

**Authors:** Jinfeng Luo, Yuling Xing, Fangzhou Li

**Affiliations:** 1 Department of Anesthesiology, West China Hospital, Sichuan University, Chengdu, Sichuan Province, P.R. China; 2 Day Surgery Center, West China Hospital, Sichuan University, Chengdu, Sichuan Province, P.R. China; Tehran University of Medical Sciences, ISLAMIC REPUBLIC OF IRAN

## Abstract

**Background:**

Previous observational studies have shown controversial results about the relationship between lipid levels and low back pain (LBP). Herein, we aimed to explore the potential causal relationship between lipid levels and LBP by using the mendelian randomization (MR) analysis.

**Methods:**

In this two-sample MR study, data were extracted from publicly available MRC Integrative Epidemiology Unit database. Three single-nucleotide polymorphisms (SNPs) of lipid levels [high density lipoprotein cholesterol (HDL-C), low density lipoprotein cholesterol (LDL-C), and triglycerides (TG)] and two SNPs of LBP risk (LBP and back pain) were retrieved and used as genetic instrumental variables. Inverse-variance weighted (IVW), weighted median, MR-Egger, robust adjusted profile score (MR-RAPS), and MR-PRESSO were used to examine the potential causal association between lipid levels and LBP.

**Results:**

IVW (fixed effect) estimation indicated that increased HDL-C level was negatively related to the odds of LBP for European populations. [odds ratio (OR) = 0.923, 95% confidence interval (CI): 0.857–0.993, *P* = 0.0323]. Similar results were also found in IVW (random effect) (OR = 0.923, 95% CI: 0.866–0.983, *P* = 0.0134), MR-Egger (OR = 0.858, 95%CI: 0.757–0.973, *P* = 0.0177), MR-RAPS (OR = 0.932, 95%CI: 0.871–0.997, *P* = 0.0419), and MR-PRESSO (OR = 0.933, 95%CI: 0.880–0.989, *P* = 0.0198) analyses. Whereas no causal link was observed between LDL-C/TG and LBP risk (*P*>0.05).

**Conclusion:**

This two-sample MR study demonstrated a causal relationship between lipid levels and LBP risk. Further investigations are necessary to elucidate the causal association and specific underlying mechanisms by which lipid levels contribute to the development of LBP.

## Introduction

Low back pain (LBP) is recognized as the primary contributor to musculoskeletal disabilities worldwide [1]. A global survey indicated that approximately 619 million individuals experienced LBP in 2020, imposing a substantial medical burden on society and seriously impacting the patients’ quality of life [2]. Identifying potential indicators linked to LBP was important for the prevention and prognostic management of this condition.

LBP may be influenced by lipid levels through the mechanisms of inflammation and atherosclerosis [3–5]. Previous several observational studies have reported a relationship between lipid levels and LBP risk, but no consistent conclusions have not yet been reached. A cohort study conducted by Leino-Arjas et al., revealed a significant association between elevated levels of total cholesterol (TC) and triglycerides (TG) with the development of radiating LBP [6]. A cross-sectional study found that TG was positively associated with LBP, and high density lipoprotein cholesterol (HDL-C) inversely associated with LBP in the Norwegian female population. However, no significant correlation was observed between total TC and LBP [7]. Similarly, Perera et al. discovered a significant association between elevated TG levels and the risk of back pain in elderly women [8]. Furthermore, a study conducted on middle-aged Japanese individuals have revealed that low HDL-C, and high low density lipoprotein cholesterol (LDL-C)/HDL-C ratio was linked to an increased susceptibility to LBP, respectively [9]. Confounding factors and reverse causation may have contributed to the inconsistent results observed in these observational studies.

Mendelian randomization (MR) analysis is a method using genetic variants as an instrumental variable to infer the causal relationship between exposure and outcome [10]. Unlike traditional observational studies, MR has the ability to avoid the reverse causality inference and enables the assessment of long-term effects of exposure on outcomes [11]. Therefore, the objective of this study is to investigate the causal association between lipid levels and LBP using MR analysis, which provided valuable insights for the prevention and prognostic management of LBP.

## Methods

### Data sources

**Fig 1:**
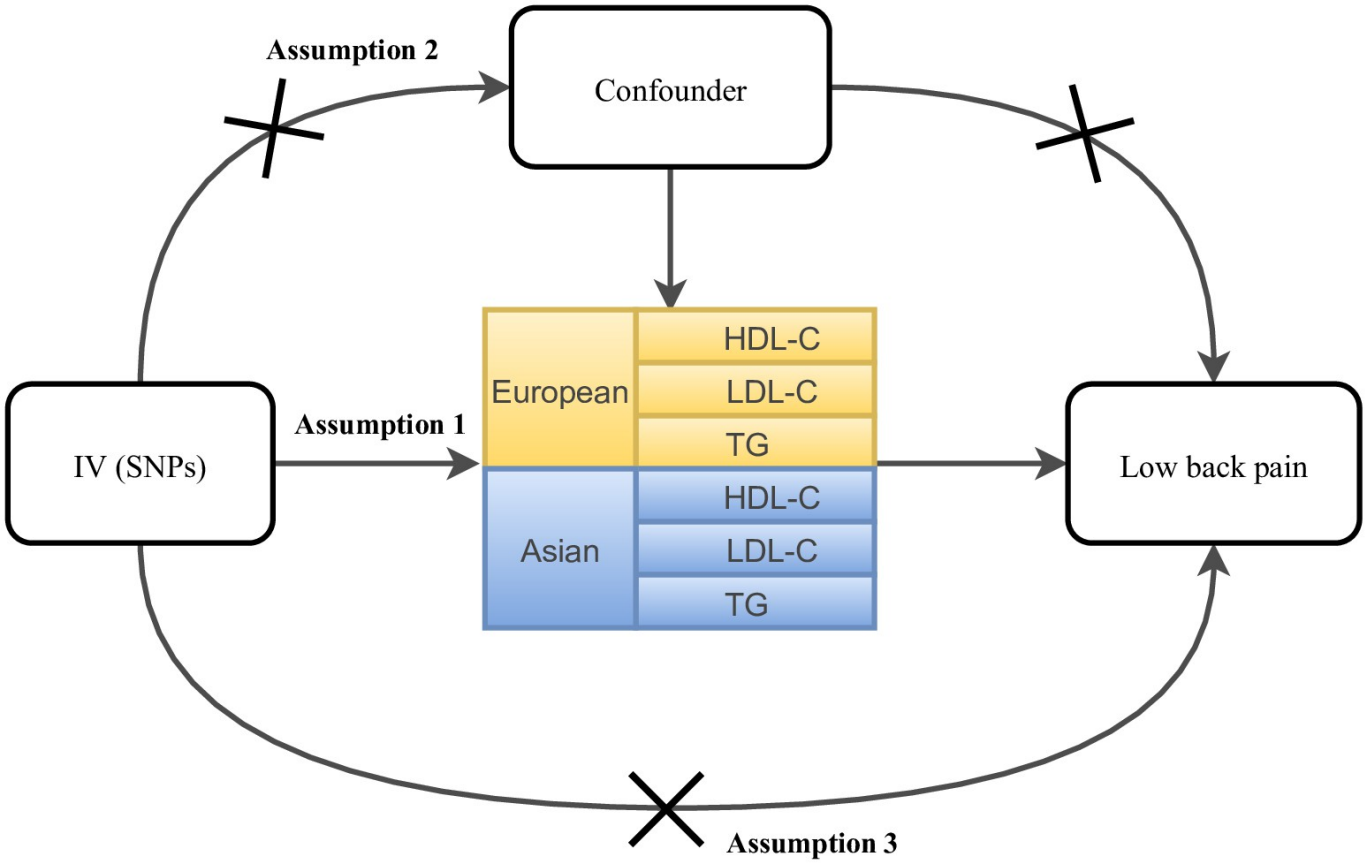
Overview of the assumptions of the mendelian randomization design.

Two-sample MR was used to assess the causal relationship between lipid levels (exposure) and LBP risk (outcome) in this study. Three assumptions were established in this study (Fig 1): assumption one-relevance assumption: the genetic variants were correlated with the exposure; assumption two-independence assumption: genetic variates were not linked to any confounder variables between the exposure and outcome; assumption three-exclusion restriction assumption: genetic variates only affect the outcome through the path of the exposure.

All data was sourced exclusively from the publicly available MRC Integrative Epidemiology Unit (IEU) database [IEU OpenGWAS project (mrcieu.ac.uk)]. Therefore, this study did not require an approval of West China Hospital, Sichuan University institutional review board. Due to the retrospective nature of the study, the West China Hospital, Sichuan University institutional review board waived the requirement for written informed consent.

### Instrumental variable selection

Table 1 summarizes single-nucleotide polymorphisms (SNPs) related to exposure and outcome. We extracted three SNPs of lipid levels (HDL-C, LDL-C, and TG) and two SNPs of LBP risk (LBP and back pain). Instrumental variables (IVs) for HDL-C [12], LDL-C [13], and TG [14] in Europeans were derived from the UK Biobank. Instrumental variables for HDL-C, LDL-C, and TG in Asian were obtained from the genome-wide association studies (GWASs) (gwas.mrcieu.ac.uk). Instrumental variables for LBP in Europeans were derived from the FINNGEN. Instrumental variables for back pain in Asian were obtained from the genome-wide association studies (GWASs) (gwas.mrcieu.ac.uk). The optimal IVs were chosen through a series of quality control measures. Initially, we employed a threshold criterion to identify the IVs associated with exposure (*P*<5 × 10^−8^). Subsequently, SNPs exhibiting linkage disequilibrium (LD) were excluded from the analysis (r^2^ = 0.001, clumping distance = 10,000 kb). In addition, any selected SNPs displaying palindromic characteristics were eliminated. Finally, the selected instrumental SNPs should show strongly association with exposure based on the assumptions of MR analysis, avoiding the presence of weak instruments bias. Thus, we calculated the F statistic and variance explained (R^2^). If the F statistic exceeds 10, it indicates small likelihood of weak instrumental variable bias [15].

**Table 1:** Summary of genetic variants associated with exposure and outcome.

GWAS ID	Trait	Year	Sample size	Searching	Population
**Exposure**					
ebi-a-GCST90014007	HDL	2021	357,810	Mbatchou J et al. [12]	European
ebi-a-GCST90002412	LDL	2020	431,167	Klimentidis YC et al. [13]	European
ieu-b-111	TG	2020	441,016	Richardson TG et al. [14]	European
ukb-e-30760_CSA	HDL	2020	7688	gwas.mrcieu.ac.uk	Asian
ukb-e-LDLC_CSA	LDL	2020	7985	gwas.mrcieu.ac.uk	Asian
ukb-e-30870_CSA	TG	2020	8415	gwas.mrcieu.ac.uk	Asian
**Outcome**					
finn-b-M13_LOWBACKPAIN	Low back pain	2021	177,860	FINNGEN	European
ukb-e-760_CSA	Back pain	2020	8876	gwas.mrcieu.ac.uk	Asian

HDL, high density lipoprotein cholesterol; LDL, low density lipoprotein; TG, triglyceride

### Horizontal pleiotropy

MR analysis should be performed on the premise of ensuring the absence of horizontal pleiotropy. The presence of horizontal pleiotropy may violate the independence assumption (assumption two) and exclusion restriction assumption (assumption three) of MR analysis. In this study, we utilized MR-Egger regression to test the horizontal pleiotropy. The significant intercept term obtained by MR-Egger implies the existence of pleiotropy.

### Statistical analysis

We utilized five popular two-sample MR analysis approaches in our study, namely inverse-variance weighted (IVW), weighted median, MR-Egger, robust adjusted profile score (MR-RAPS), and MR-PRESSO, to analyze the association between lipid levels and LBP risk. IVW method can offer a relatively accurate causal assessment by amalgamating Wald estimates for each IV through a meta-analysis approach, which was the main methodology employed in this study to generate estimates of causal effect [16]. We employed both fixed and random effects models for the IVW test, where odds ratios (ORs) along with 95% confidence intervals (CIs) were utilized to quantify the effect size. Weighted median can provide robust estimate of effect when more than 50% of the IVs are invalid, and it has the potential to reduce the type I error and precisely assess causal association if horizontal pleiotropy exists [17]. The MR-PRESSO method was used to detect horizontal pleiotropy and evaluate the presence of pleiotropic outliers. In this study, the presence of a horizontal pleiotropic effect is suggested when the *P*<0.05 of the intercept in the MR-Egger test or when the *P*<0.05 of the observed residual sum of squares (RSS_obs_) in the MR-PRESSO test [18]. The heterogeneity between IVs was assessed using Cochrane’s Q-statistic [19]. A significance level of *P*<0.05 indicates the presence of significant heterogeneity. The leave-one-out sensitivity test was employed to assess the sensitivity of a single SNP by excluding IVs one by one [20].

Using R software to perform all statistical analyses for this study, and the R package “TwoSampleMR” was utilized to conduct MR analysis. *P*< 0.05 was set as statistical significance.

## Results

### Instrumental variables selection

After screening, we identified 305 HDL-C related SNPs, 322 LDL-C related SNPs, and 274 related TG in European populations. 18 SNPs for Asian populations, which comprised six HDL-C, three LDL-C, and nine TG. As shown in Table 2, the F-statistics for each SNP exposure exceeded 10, demonstrating a low possibility of weak instrumental variable bias. In addition, the results of MR-Egger and MR-PRESSO analyses showed that horizontal pleiotropy was not present in all selected SNPs (*P*>0.05). Simultaneously, the heterogeneity testing results also indicated that there was no significant heterogeneity in all identified SNPs (*P*>0.05).

**Table 2:** Heterogeneity and horizontal pleiotropy of instrumental variables.

Exposure	Strength			Pleiotropy Test				Heterogeneity Test			
				MR-Egger		MR-PRESSO					
	N	F value	R^2^ (%)	Intercept	P	RSS_obs_	P	MR-Egger (Q)	P	IVW (Q)	P
**European**											
HDL	305	134.945	0.038	0.0024	0.1626	231.7586	0.998	198.5497	0.9994	200.5099	0.9992
LDL	322	160.876	0.037	0.0029	0.1132	241.3295	1.000	188.3225	0.9999	190.8477	0.9998
TG	274	133.389	0.030	0.0001	0.9674	228.6895	0.974	198.5842	0.9504	198.5858	0.9551
**Asian**											
HDL	6	71.881	0.927	0.1135	0.2951	5.9156	0.568	2.8122	0.5897	4.2609	0.5125
LDL	3	99.452	1.230	-0.2391	0.3193	-	-	0.4171	0.5184	3.744	0.1538
TG	9	75.01	0.884	-0.0103	0.865	3.1737	0.978	2.7102	0.9105	2.7413	0.9495

N, number of instrument variables; IVW, inverse-variance weight; MR, Mendelian randomization; RSS_obs_, residual sum of squares; HDL, high density lipoprotein cholesterol; LDL, low density lipoprotein; TG, triglyceride

### Two-sample MR analysis for causal association between lipid levels and LBP

**Fig 2:**
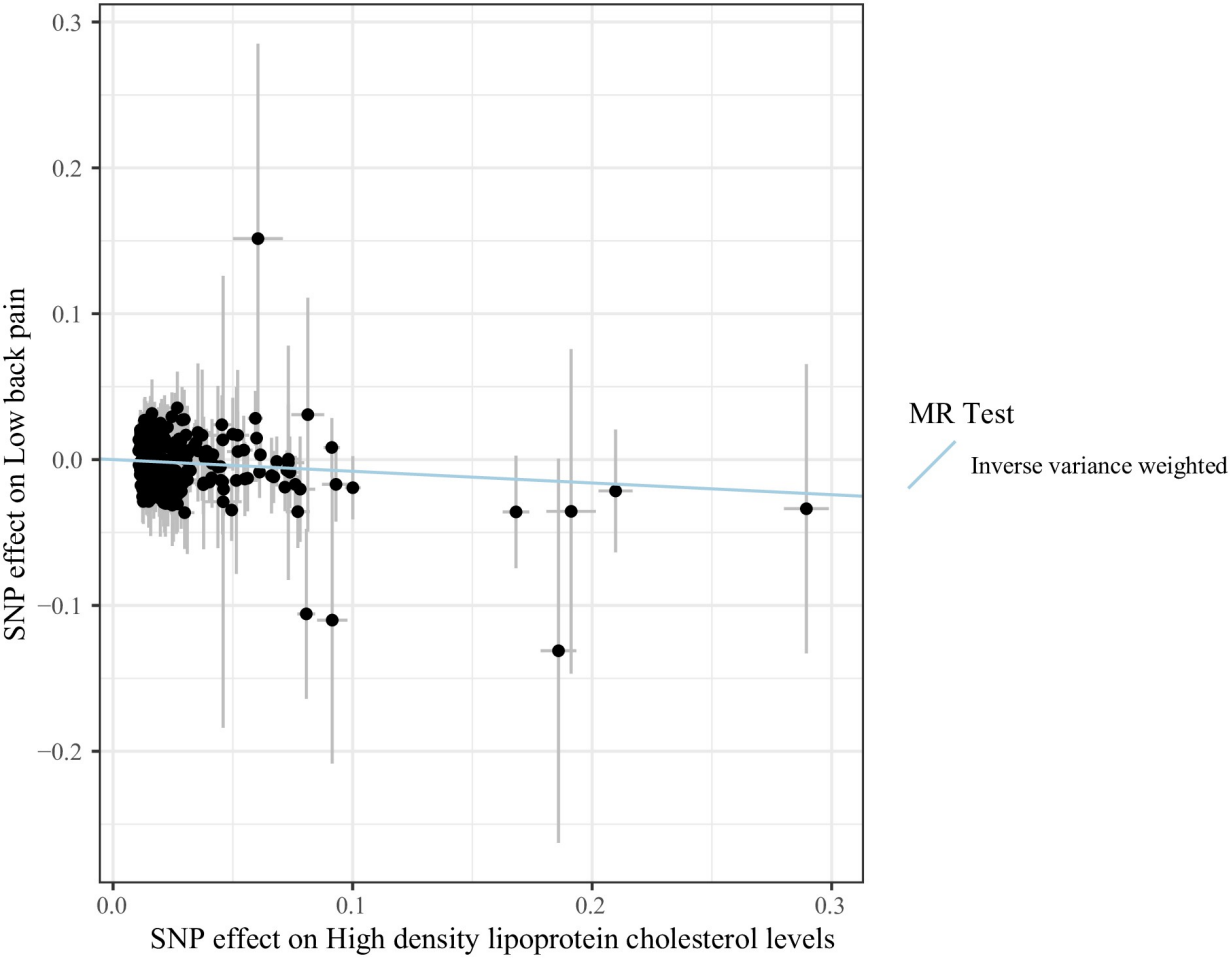
Scatter plot of the causality of lipid levels with low back pain. The slopes of line represent the causal effect of each method, respectively.

**Fig 3:**
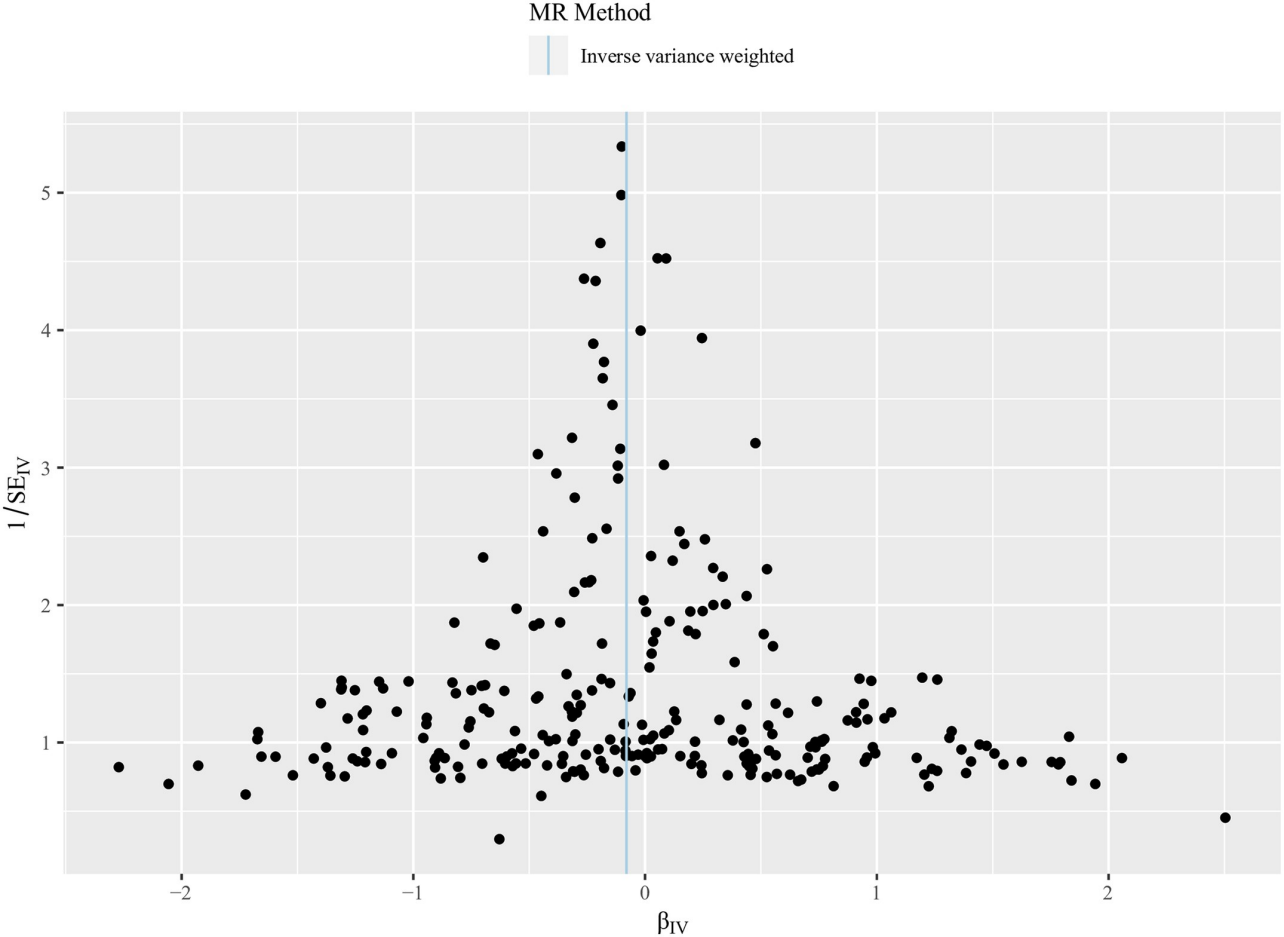
Funnel plot to detect whether the observed association was along with obvious heterogeneity.

**Fig 4:**
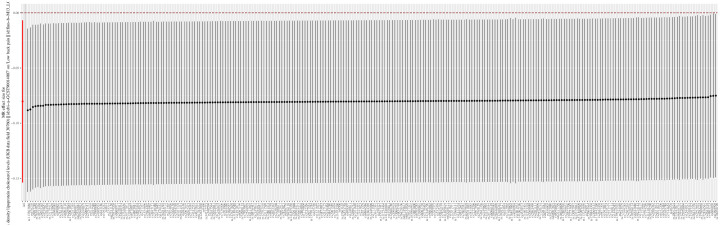
Leave-one-out sensitivity analysis to evaluate whether any individual instrumental variable was driving this causal effect.

Table 3 shows the causal relationship between lipid levels and LBP risk. The results suggested that the increased relative abundance of HDL-C was negatively related to the odds of LBP, which was significant for European populations in the IVW (random effect) (OR = 0.923, 95%CI: 0.866–0.983, *P* = 0.0134), IVW (fixed effect) (OR = 0.923, 95%CI: 0.857–0.993, *P* = 0.0323), MR-Egger (OR = 0.858, 95%CI: 0.757–0.973, *P* = 0.0177), MR-RAPS (OR = 0.932, 95%CI: 0.871–0.997, *P* = 0.0419), and MR-PRESSO (OR = 0.933, 95%CI: 0.880–0.989, *P* = 0.0198). The scatter plot (Fig 2) and the funnel plot (Fig 3) also further present these causal estimates. Meanwhile, Fig 4 demonstrates that the causal relationship between lipid levels and LBP was not caused by any individual SNP by using leave-one-out sensitivity analysis.

**Table 3:** Two-sample MR analysis for causal association between lipid levels and low back pain.

Exposure	Method	OR (95%CI)	P	FDR correction
**European**				
HDL	MR-Egger	0.858 (0.757–0.973)	0.0177	0.0396
	Weighted median	0.899 (0.801–1.008)	0.0681	0.0681
	IVW (Random effect)	0.923 (0.866–0.983)	0.0134	0.0396
	IVW (Fixed effect)	0.923 (0.857–0.993)	0.0323	0.0485
	MR-RAPS	0.932 (0.871–0.997)	0.0419	0.0503
	MR-PRESSO	0.933 (0.880–0.989)	0.0198	0.0396
LDL	MR Egger	0.939 (0.834–1.058)	0.3017	
	Weighted median	0.987 (0.884–1.101)	0.8094	
	IVW (Random effect)	1.016 (0.958–1.077)	0.6049	
	IVW (Fixed effect)	1.016 (0.948–1.089)	0.6607	
	MR-RAPS	1.001 (0.940–1.066)	0.9648	
	MR-PRESSO	1.001 (0.949–1.057)	0.9593	
TG	MR Egger	1.025 (0.919–1.144)	0.6570	
	Weighted median	1.086 (0.964–1.222)	0.1737	
	IVW (Random effect)	1.027 (0.961–1.098)	0.4320	
	IVW (Fixed effect)	1.027 (0.956–1.104)	0.4691	
	MR-RAPS	1.011 (0.943–1.083)	0.7630	
	MR-PRESSO	1.011 (0.949–1.076)	0.7406	
**Asian**				
HDL	MR Egger	0.494 (0.150–1.619)	0.3088	
	Weighted median	0.867 (0.524–1.435)	0.5778	
	IVW (Random effect)	0.975 (0.659–1.445)	0.9012	
	IVW (Fixed effect)	0.975 (0.637–1.493)	0.9088	
	MR-RAPS	0.975 (0.633–1.503)	0.9093	
	MR-PRESSO	0.975 (0.659–1.445)	0.9061	
LDL	MR Egger	2.805 (1.022–7.696)	0.2949	
	Weighted median	1.242 (0.729–2.117)	0.4247	
	IVW (Random effect)	1.210 (0.653–2.241)	0.5443	
	IVW (Fixed effect)	1.210 (0.771–1.898)	0.4068	
	MR-RAPS	1.213 (0.770–1.911)	0.4056	
	MR-PRESSO	-	-	
TG	MR Egger	0.918 (0.419–2.011)	0.8373	
	Weighted median	0.887 (0.591–1.333)	0.5641	
	IVW (Random effect)	0.861 (0.711–1.043)	0.1253	
	IVW (Fixed effect)	0.861 (0.621–1.194)	0.3696	
	MR-RAPS	0.861 (0.617–1.200)	0.3767	
	MR-PRESSO	0.861 (0.711–1.043)	0.1639	

OR, odds ratios; CI, confidence interval; HDL, high density lipoprotein cholesterol; LDL, low density lipoprotein; TG, triglyceride; IVW, inverse-variance weight; MR, Mendelian randomization.

## Discussion

In the present study, we performed a two-sample MR analysis to explore the potential causal relationship between lipid levels and LBP risk using summary statistics from published GWAS. Our findings demonstrated a causal association between HDL-C and LBP risk in European populations, whereas no causal link was observed between LDL-C/TG and LBP risk. These results have important implications for understanding the effect of HDL on the development of LBP and can provide valuable insights for preventing and managing LBP.

Recently, MR approach is widely employed to ascertain potential causal relationships between exposure factors and outcomes [21]. By circumventing the influence of confounding variables and reverse causality, MR analysis method provides a more robust estimate of causality compared to traditional observational studies [22]. Li W et al., found that elevated interleukin 6 level was associated with a reduced the risk of LBP by using MR approach [5]. Jiang X et al., performed two-sample MR study to assess the causal effect between serum 25-hydroxyvitamin D level and LBP risk. Their results showed that an elevated genetic level of serum 25-hydroxyvitamin D level was found to be related to a decreased risk of experiencing LBP in the European population [23]. To the best of our knowledge, existing observational studies have reported the association regarding lipid levels and LBP risk, but the results have been inconsistent. The current evidence is insufficient to establish a causal relationship between lipid levels and LBP risk. Therefore, in this study, we utilized five popular two-sample MR analysis approaches to analyze the causal association between lipid levels and LBP risk. By IVW analysis, we found that HDL-C was negatively related to the odds of LBP in European populations, but there was no causal link between LDL-C /TG and LBP risk. This present study is also the first MR analysis of lipid levels for LBP development. Compared with previous studies, our study offers more robust evidence supporting the association between lipid levels and the risk of developing LBP in European populations.

In fact, the underlying mechanisms of HDL-C and LBP risk are unclear. The degeneration of lumbar discs is a significant contributor to the occurrence of LBP [24]. Previous research has indicated a potential association between lumbar disc degeneration and the development of atherosclerosis [25]. HDL is a cholesterol transporter, and exhibits various functional properties, including antioxidant, anti-inflammatory, and immune-regulatory activities [26]. In a study of Bandeali S et al., they also pointed out that HDL-C level was inversely linked with the risk for the development of atherosclerosis [27]. In addition, elevated lipid levels may contribute to an upregulation of the inflammatory response and systemic inflammation, thereby potentially leading to the degeneration of lumbar discs [28]. Specific underlying mechanisms regarding the association of lipid levels with the development of LBP still require further investigation.

Overall, this study first explored the potential causal association between lipid levels and LBP risk by employing two-sample MR analysis. The findings may provide genetic evidence for the contentious findings regarding the relationship of lipid levels and LBP risk, emphasizing the importance of managing lipid profiles comprehensively in order to improve LBP control.

Nevertheless, there were some limitations in this study. First, due to the limited GWAS data from Asian populations, the number of IVs reflecting outcomes and exposure is relatively limited. Second, the existence of a linear association between lipid levels and back pain requires further investigation, and the possibility of a threshold effect cannot be disregarded. Third, the potential causal relationship between lipid levels and LBP risk was found to be limited in European populations, necessitating further research to ascertain the generalizability of these findings to other populations.

## Conclusion

In short, our study provided genetic evidence that high HDL-C may reduce the risk of LBP in European populations, whereas there was no statistically significant relationship between LDL-C/TG and LBP risk. HDL-C may become novel biomarkers and provide insights for the development of effective strategies for preventing and managing LBP. However, further investigations are necessary to elucidate the causal association and specific underlying mechanisms by which lipid levels contribute to the development of LBP.

## References

[1] Sany SA, Tanjim T, Hossain MI. Low back pain and associated risk factors among medical students in Bangladesh: a cross-sectional study. F1000Res. 2021;10:698. Epub 2021/07/30. doi: 10.12688/f1000research.55151.3 . 35999897 PMC9360907

[2] Global, regional, and national burden of low back pain, 1990–2020, its attributable risk factors, and projections to 2050: a systematic analysis of the Global Burden of Disease Study 2021. Lancet Rheumatol. 2023;5(6):e316–e29. Epub 2023/06/05. doi: 10.1016/S2665-9913(23)00098-X . 37273833 PMC10234592

[3] Hulthe J, Fagerberg B. Circulating oxidized LDL is associated with subclinical atherosclerosis development and inflammatory cytokines (AIR Study). Arterioscler Thromb Vasc Biol. 2002;22(7):1162–7. Epub 2002/07/16. doi: 10.1161/01.atv.0000021150.63480.cd . 12117732

[4] Farrell SF, Sterling M, Klyne DM, Mustafa S, Campos AI, Kho PF, et al. Genetic impact of blood C-reactive protein levels on chronic spinal & widespread pain. Eur Spine J. 2023;32(6):2078–85. Epub 2023/04/18. doi: 10.1007/s00586-023-07711-7 . 37069442

[5] Li W, Lu Q, Qian J, Feng Y, Luo J, Luo C, et al. Assessing the causal relationship between genetically determined inflammatory biomarkers and low back pain risk: a bidirectional two-sample Mendelian randomization study. Front Immunol. 2023;14:1174656. Epub 2023/07/31. doi: 10.3389/fimmu.2023.1174656 . 37520547 PMC10372790

[6] Leino-Arjas P, Kaila-Kangas L, Solovieva S, Riihimäki H, Kirjonen J, Reunanen A. Serum lipids and low back pain: an association? A follow-up study of a working population sample. Spine (Phila Pa 1976). 2006;31(9):1032–7. Epub 2006/04/28. doi: 10.1097/01.brs.0000214889.31505.08 . 16641781

[7] Heuch I, Heuch I, Hagen K, Zwart JA. Associations between serum lipid levels and chronic low back pain. Epidemiology. 2010;21(6):837–41. Epub 2010/08/28. doi: 10.1097/EDE.0b013e3181f20808 . 20798637

[8] Perera RS, Chen L, Ferreira ML, Arden NK, Radojčić MR, Kluzek S. Age- and sex-specific effects of obesity, metabolic syndrome and its components on back pain: The English Longitudinal Study of Ageing. Joint Bone Spine. 2022;89(5):105366. Epub 2022/03/02. doi: 10.1016/j.jbspin.2022.105366 . 35227920

[9] Yoshimoto T, Ochiai H, Shirasawa T, Nagahama S, Kobayashi M, Minoura A, et al. Association between serum lipids and low back pain among a middle-aged Japanese population: a large-scale cross-sectional study. Lipids Health Dis. 2018;17(1):266. Epub 2018/11/27. doi: 10.1186/s12944-018-0907-1 . 30474551 PMC6260842

[10] Ho J, Mak CCH, Sharma V, To K, Khan W. Mendelian Randomization Studies of Lifestyle-Related Risk Factors for Osteoarthritis: A PRISMA Review and Meta-Analysis. Int J Mol Sci. 2022;23(19). Epub 2022/10/15. doi: 10.3390/ijms231911906 . 36233208 PMC9570129

[11] Bowden J, Holmes MV. Meta-analysis and Mendelian randomization: A review. Res Synth Methods. 2019;10(4):486–96. Epub 2019/03/13. doi: 10.1002/jrsm.1346 . 30861319 PMC6973275

[12] Mbatchou J, Barnard L, Backman J, Marcketta A, Kosmicki JA, Ziyatdinov A, et al. Computationally efficient whole-genome regression for quantitative and binary traits. Nat Genet. 2021;53(7):1097–103. Epub 2021/05/22. doi: 10.1038/s41588-021-00870-7 . 34017140

[13] Klimentidis YC, Arora A, Newell M, Zhou J, Ordovas JM, Renquist BJ, et al. Phenotypic and Genetic Characterization of Lower LDL Cholesterol and Increased Type 2 Diabetes Risk in the UK Biobank. Diabetes. 2020;69(10):2194–205. Epub 2020/06/05. doi: 10.2337/db19-1134 . 32493714 PMC7506834

[14] Richardson TG, Sanderson E, Palmer TM, Ala-Korpela M, Ference BA, Davey Smith G, et al. Evaluating the relationship between circulating lipoprotein lipids and apolipoproteins with risk of coronary heart disease: A multivariable Mendelian randomisation analysis. PLoS Med. 2020;17(3):e1003062. Epub 2020/03/24. doi: 10.1371/journal.pmed.1003062 . 32203549 PMC7089422

[15] Long Y, Tang L, Zhou Y, Zhao S, Zhu H. Causal relationship between gut microbiota and cancers: a two-sample Mendelian randomisation study. BMC Med. 2023;21(1):66. Epub 2023/02/23. doi: 10.1186/s12916-023-02761-6 . 36810112 PMC9945666

[16] Gui L, He X, Tang L, Yao J, Pi J. Obesity and head and neck cancer risk: a mendelian randomization study. BMC Med Genomics. 2023;16(1):200. Epub 2023/08/25. doi: 10.1186/s12920-023-01634-4 . 37620971 PMC10463997

[17] Bowden J, Davey Smith G, Haycock PC, Burgess S. Consistent Estimation in Mendelian Randomization with Some Invalid Instruments Using a Weighted Median Estimator. Genet Epidemiol. 2016;40(4):304–14. Epub 2016/04/12. doi: 10.1002/gepi.21965 . 27061298 PMC4849733

[18] Verbanck M, Chen CY, Neale B, Do R. Detection of widespread horizontal pleiotropy in causal relationships inferred from Mendelian randomization between complex traits and diseases. Nat Genet. 2018;50(5):693–8. Epub 2018/04/25. doi: 10.1038/s41588-018-0099-7 . 29686387 PMC6083837

[19] Zhao H, Zhu J, Ju L, Sun L, Tse LA, Kinra S, et al. Osteoarthritis & stroke: a bidirectional mendelian randomization study. Osteoarthritis Cartilage. 2022;30(10):1390–7. Epub 2022/07/08. doi: 10.1016/j.joca.2022.06.006 . 35798177

[20] Cao Z, Wu Y, Li Q, Li Y, Wu J. A causal relationship between childhood obesity and risk of osteoarthritis: results from a two-sample Mendelian randomization analysis. Ann Med. 2022;54(1):1636–45. Epub 2022/06/16. doi: 10.1080/07853890.2022.2085883 . 35703935 PMC9225762

[21] Lv Z, Cui J, Zhang J. Smoking, alcohol and coffee consumption and risk of low back pain: a Mendelian randomization study. Eur Spine J. 2022;31(11):2913–9. Epub 2022/09/17. doi: 10.1007/s00586-022-07389-3 . 36114324

[22] Qian Y, He Z, Zhao SS, Liu B, Chen Y, Sun X, et al. Genetically Determined Circulating Levels of Cytokines and the Risk of Rheumatoid Arthritis. Front Genet. 2022;13:802464. Epub 2022/02/25. doi: 10.3389/fgene.2022.802464 . 35198006 PMC8859847

[23] Jiang X, Zhou R, He Y, Zhu T, Zhang W. Causal effect of serum 25-hydroxyvitamin D levels on low back pain: A two-sample mendelian randomization study. Front Genet. 2022;13:1001265. Epub 2022/10/11. doi: 10.3389/fgene.2022.1001265 . 36212121 PMC9534573

[24] Kirnaz S, Capadona C, Wong T, Goldberg JL, Medary B, Sommer F, et al. Fundamentals of Intervertebral Disc Degeneration. World Neurosurg. 2022;157:264–73. Epub 2021/12/22. doi: 10.1016/j.wneu.2021.09.066 . 34929784

[25] Keser N, Celikoglu E, İs M, İlgezdi ZD, Sunar B, Aydin YS, et al. Is there a relationship between blood lipids and lumbar disc herniation in young Turkish adults? Arch Med Sci Atheroscler Dis. 2017;2(1):e24–e8. Epub 2017/09/15. doi: 10.5114/amsad.2017.68651 . 28905044 PMC5596115

[26] von Eckardstein A, Nordestgaard BG, Remaley AT, Catapano AL. High-density lipoprotein revisited: biological functions and clinical relevance. Eur Heart J. 2023;44(16):1394–407. Epub 2022/11/08. doi: 10.1093/eurheartj/ehac605 . 36337032 PMC10119031

[27] Bandeali S, Farmer J. High-density lipoprotein and atherosclerosis: the role of antioxidant activity. Curr Atheroscler Rep. 2012;14(2):101–7. Epub 2012/03/24. doi: 10.1007/s11883-012-0235-2 . 22441969

[28] Zhang Y, Zhao Y, Wang M, Si M, Li J, Hou Y, et al. Serum lipid levels are positively correlated with lumbar disc herniation—a retrospective study of 790 Chinese patients. Lipids Health Dis. 2016;15:80. Epub 2016/04/20. doi: 10.1186/s12944-016-0248-x . 27090514 PMC4836107

